# Activation of sterol regulatory element‐binding protein 1 (SREBP1)‐mediated lipogenesis by the Epstein–Barr virus‐encoded latent membrane protein 1 (LMP1) promotes cell proliferation and progression of nasopharyngeal carcinoma

**DOI:** 10.1002/path.5130

**Published:** 2018-08-22

**Authors:** Angela Kwok‐Fung Lo, Raymond Wai‐Ming Lung, Christopher W Dawson, Lawrence S Young, Chuen‐Wai Ko, Walter Wai Yeung, Wei Kang, Ka‐Fai To, Kwok‐Wai Lo

**Affiliations:** ^1^ Department of Anatomical and Cellular Pathology, Li Ka Shing Institute of Health Science, Chinese University of Hong Kong Prince of Wales Hospital Shatin Hong Kong; ^2^ Institute of Cancer & Genomic Science, Cancer Research UK Cancer Centre University of Birmingham Birmingham UK; ^3^ Warwick Medical School University of Warwick Coventry UK

**Keywords:** nasopharyngeal carcinoma, Epstein–Barr virus, lipogenesis, LMP1, SREBP1

## Abstract

Nasopharyngeal carcinoma (NPC) is closely associated with Epstein–Barr virus (EBV) infection. The EBV‐encoded latent membrane protein 1 (LMP1), which is commonly expressed in NPC, engages multiple signaling pathways that promote cell growth, transformation, and metabolic reprogramming. Here, we report a novel function of LMP1 in promoting *de novo* lipogenesis. LMP1 increases the expression, maturation and activation of sterol regulatory element‐binding protein 1 (SREBP1), a master regulator of lipogenesis, and its downstream target fatty acid synthase (FASN). LMP1 also induces *de novo* lipid synthesis and lipid droplet formation. In contrast, small interfering RNA (siRNA) knockdown of LMP1 in EBV‐infected epithelial cells diminished SREBP1 activation and lipid biosynthesis. Furthermore, inhibition of the mammalian target of rapamycin (mTOR) pathway, through the use of either mTOR inhibitors or siRNAs, significantly reduced LMP1‐mediated SREBP1 activity and lipogenesis, indicating that LMP1 activation of the mTOR pathway is required for SREBP1‐mediated lipogenesis. In primary NPC tumors, FASN overexpression is common, with high levels correlating significantly with LMP1 expression. Moreover, elevated FASN expression was associated with aggressive disease and poor survival in NPC patients. Luteolin and fatostatin, two inhibitors of lipogenesis, suppressed lipogenesis and proliferation of nasopharyngeal epithelial cells, effects that were more profound in cells expressing LMP1. Luteolin and fatostatin also dramatically inhibited NPC tumor growth *in vitro* and *in vivo*. Our findings demonstrate that LMP1 activation of SREBP1‐mediated lipogenesis promotes tumor cell growth and is involved in EBV‐driven NPC pathogenesis. Our results also reveal the therapeutic potential of utilizing lipogenesis inhibitors in the treatment of locally advanced or metastatic NPC. © 2018 The Authors. *The Journal of Pathology* published by John Wiley & Sons Ltd on behalf of Pathological Society of Great Britain and Ireland.

## Introduction

Non‐keratinizing undifferentiated nasopharyngeal carcinoma (NPC) is a distinct type of cancer that is prevalent in Southeast Asia and southern China. The unique feature of NPC is its strong association with Epstein–Barr virus (EBV) infection 1, 2. Among the EBV‐encoded gene products expressed in NPC, latent membrane protein 1 (LMP1) is of particular interest, as it shows oncogenic properties *in vitro* and *in vivo*. LMP1 is an integral membrane protein containing two signaling domains: CTAR1 and CTAR2. Through these two domains, LMP1 engages multiple signaling cascades that include the Ras–extracellular signal‐regulated kinase (ERK)–mitogen‐activated protein kinase (MAPK), phosphoinositide 3‐kinase (PI3K)–AKT, nuclear factor‐κB (NF‐κB) and p38‐MAPK pathways, which modulate the expression of a variety of cellular targets that contribute to the transforming activities of LMP1 1, 2, 3. Previous studies have established a role for LMP1 in promoting cell proliferation, transformation, cell invasion and migration, aerobic glycolysis and metabolic reprogramming in nasopharyngeal epithelial cells 3, 4, 5. These observations imply an essential role for LMP1 in the pathogenesis of NPC.

Deregulated lipid metabolism is an established hallmark of cancer. Cells obtain fatty acids either from the diet or through *de novo* lipid synthesis (lipogenesis). Normal cells rely primarily on dietary fatty acid for the synthesis of new structural lipids, and lipogenesis is not universal. However, cancer cells extensively engage *de novo* lipogenesis to produce long‐chain fatty acids that are essential for the synthesis of glycerophospholipid membrane and membrane signal molecules during rapid cell proliferation (supplementary material, Figure S5). Fatty acids are also necessary for energy storage as lipid droplets 6, 7, 8. Lipogenesis is tightly regulated by sterol regulatory element‐binding protein (SREBP) 1, a transcription factor that regulates the transcription of most genes involved in lipogenesis 9, 10, 11. There are two SREBP1 isoforms (SREBP1a and SREBP1c) encoded by *SREBF1*. SREBF1a and SREBF1c are produced from different promoters, differing only in the lengths of their N‐terminal transactivation domains. SREBPs are synthesized as precursor proteins bound to the endoplasmic reticulum (ER) membrane. After stimulation, the SREBP precursor undergoes proteolytic cleavage in the Golgi to release the transcriptionally active N‐terminal domain. Once mature, active SREBP1 translocates to the nucleus, where it binds to sterol regulatory elements (SREs) within its promoter and its target genes 9, 10, 11. Mammalian target of rapamycin (mTOR) complex 1 (mTORC1) and mTOR complex 2 (mTORC2) have been shown to regulate SREBP1 activity and lipogenesis. Activation of mTOR signaling by the Ras–ERK and PI3K–AKT pathways increases expression of SREBPs and SREBP‐mediated lipogenesis 12, 13, 14. Numerous lipogenic genes, including that encoding fatty acid synthase (FASN), are upregulated in a variety of cancers 7, 8, 10. In this study, we demonstrate that FASN expression is common in primary NPC tumors, with higher levels correlating with LMP1 expression. Furthermore, LMP1 activates *de novo* lipogenesis, and LMP1 activation of SREBP1‐mediated lipogenesis contributes to cancer cell growth and tumor progression. These findings imply the involvement of LMP1‐mediated lipogenesis in the pathogenesis of EBV‐infected NPC.

## Materials and methods

### Cell lines, chemicals, and pharmacological inhibitors

C666‐1 and HK‐1 NPC cell lines were maintained in RPMI‐1640 medium supplemented with 10% fetal bovine serum. The SV40 large T‐immortalized nasopharyngeal epithelial cell line NP69 was maintained in keratinocyte serum‐free medium (Thermo Fisher Scientific, Waltham, MA, USA). Torin 1, Torin 2, luteolin and fatostatin were obtained from Abcam (Cambridge, UK). Further details are presented in supplementary material, Supplementary materials and methods.

### DNA constructs and small interfering RNA (siRNA)

Scrambled short hairpin RNA (shRNA) and LMP1 shRNA vectors were generated by inserting a fragment of synthesized oligonucleotide with a scrambled sequence or a sequence for LMP1 into pSUPER.retro.puro vector (OligoEngine, Seattle, WA, USA). The pGL2‐3xSRE luciferase vector was obtained from ATCC (Manassas, VA, USA). pGL3‐FASN was kindly provided by Qiang Liu (University of Saskatchewan, Saskatoon, Canada) 15. All siRNAs were purchased from Dharmacon (Lafayette, CO, USA). Transient transfection of siRNA and DNA was performed with Lipofectamine 2000 (Invitrogen, Carlsbad, CA, USA) and Fugene HD (Promega, Madison, WI, USA) respectively. See supplementary material, Supplementary materials and methods, for additional details.

### Western blotting analysis

Total cell lysates (5–50 μg of protein) were separated by 10% or 4–12% sodium dodecylsulfate polyacrylamide gel electrophoresis, and transferred to poly(vinylidene difluoride) membranes prior to immunoblotting. Antibodies against LMP1 (clones CS1‐4; 1:1000 dilution) were purchased from Dako (Glostrup, Denmark), and antibodies against α‐tubulin (Cat. No. sc‐8035; 1:5000 dilution) were purchased from Santa Cruz (Dallas, TX, USA). The anti‐SREBP1 antibody (2A4; 1:1000 dilution) was purchased from Abcam and Santa Cruz. All other antibodies were purchased from Cell Signaling Technology (Beverley, MA, USA). Additional details are provided in supplementary material, Supplementary materials and methods.

### Reverse transcription quantitative polymerase chain reaction (RT‐qPCR)

All RT‐qPCR products were amplified with the Power SYBR green PCR Master Mix Kit (Thermo Fisher Scientific). Details, including primer sequences, are provided in supplementary material, Supplementary materials and methods.

### Luciferase reporter assay

**Figure 1:**
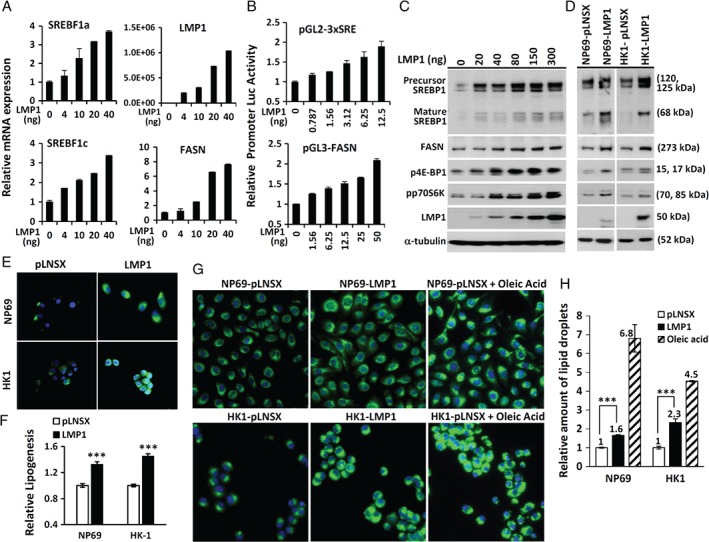
Induction of SREBP1 expression and activity by LMP1. (A) NP69 cells transfected with increasing amounts of an LMP1 expression vector as indicated were subjected to RT‐qPCR analysis for *SREBF1a*, *SREBF1c*, *LMP1*, and *FASN*. The mRNA expression of the target gene of interest was normalized to expression of *TBP*. Relative mRNA levels were calculated with the sample without the LMP1 expression vector (set at 1). (B) HEK‐293 cells were transfected with various amounts of an LMP1 expression vector together with luciferase promoter vector: pGL2‐3xSRE or pGL3‐FASN. After serum deprivation for 12 h, cells were harvested for reporter activity analysis. The firefly luciferase activity was normalized to *Renilla* luciferase activity, and plotted relative to the sample without the LMP1 expression vector (set at 1). (C and D) NP69 cells transfected with increasing amounts of an LMP1 expression vector as indicated (C) or NP69 and HK1 cells stably expressing LMP1 (D) were incubated in serum‐free medium for 12 h, prior to immunoblotting analysis. (E) Immunofluorescence staining of FASN. (F) [^14^C]Acetate incorporation assay for the measurement of *de novo* lipid synthesis. (G) Nile Red fluorescence staining for lipid droplets and counterstaining with Hoechst 33342 to stain cell nuclei. As a positive control for lipid droplet staining (right panel), pLNSX control cells were incubated in medium supplemented with oleic acid for 12 h prior to staining. Nile Red fluorescence (excitation, 385 nm; emission, 535 nm) and Hoechst 33342 fluorescence (excitation, 355 nm; emission, 460 nm). (H) The relative amount of lipid droplet formation was calculated by normalizing the Hoechst 33342 fluorescence to the Nile Red signal in each well, and compared with the pLNSX vector control sample (set at 1). Data represent the means of eight determinations. Mean and standard deviation. ***p < 0.001.

Ten thousand HeLa or HEK‐293 cells grown in 96‐well plates were co‐transfected with 20 ng of the luciferase reporter construct together with increasing amounts of an LMP1 expression vector (pCDNA3‐LMP1), as indicated in Figure 1. *Renilla* pRL‐SV40 vector was transfected as an internal control to correct for transfection efficiency. Two days after transfection, cells were lysed in reporter lysis buffer, and then assayed for firefly and *Renilla* luciferase activities with the Dual‐Luciferase Reporter Assay System (Promega).

### Immunofluorescence staining

Immunofluorescence staining was performed as previously described 16 and as further outlined in supplementary material, Supplementary materials and methods.

### Lipid droplet fluorescence staining

Nile Red fluorescence staining was assessed with the Lipid Droplets Fluorescence Assay Kit according to the manufacturer's protocol (Cayman Chemical, Ann Arbor, MI, USA). Details are provided in supplementary material, Supplementary materials and methods.

### Immunohistochemical staining

Immunohistochemical staining was performed as described previously 17. Information regarding normal and tumor specimens, staining and intensity score methods are outlined in supplementary material, Supplementary materials and methods.

### Cell proliferation assay

Cell proliferation assays were performed with cell proliferation reagent CCK‐8 (Dojindo Molecular Technologies, Rockville, MD, USA) and as described in supplementary material, Supplementary materials and methods.

### *De novo* lipogenesis assay

For the lipogenesis assay, cells were incubated in serum‐free medium containing 2.5 μCi/ml [1‐^14^C]acetate for 8–12 h. After being washed with phosphate‐buffered saline (PBS), cells were lysed in 0.5% Triton X‐100/PBS. Lipids were extracted by successive addition of methanol and chloroform, followed by centrifugation at 16,000 × *g* for 15 minutes. The organic phase was air‐dried and resuspended in chloroform for scintillation counting. Results were normalized to total protein content. Details are provided in supplementary material, Supplementary materials and methods.

### *In vivo* tumorigenicity experiments

The University Animal Experimentation Ethics Committee of the Chinese University of Hong Kong approved the study protocol. In brief, C666‐1 cells (1 × 10^7^) were inoculated subcutaneously into BALB/c nude mice. When the size of tumors reached 50 mm^3^, mice were injected intravenously with PBS (vehicle), luteolin or fatostatin every 2–3 days for 3 weeks. At the endpoint, mice were killed and tumors were harvested. For further details, see supplementary material, Supplementary materials and methods.

### Statistical analysis

Statistical analyses were performed with GraphPad Prism 5.0 (GraphPad Software, La Jolla, CA, USA). *P* values were calculated with either Fisher's exact test or an unpaired two‐tailed Student's *t*‐test. The IC_50_ values of inhibitors were calculated by applying the four‐parameter logistic equation to generate the sigmoidal dose–response (variable slope) curves. Survival curves were analyzed with the Kaplan–Meier method, and were compared by use of a log‐rank test. A *P* value of <0.05 was considered to be statistically significant.

## Results

### LMP1 increases SREBP1 expression and activity

To examine the impact of LMP1 on SREBP1‐mediated lipogenesis, increasing amounts of an LMP1 expression vector were transfected into the nasopharyngeal epithelial cell line NP69. Under serum‐deprived conditions, dose‐dependent induction of *SREBF1a* and *SREBF1c* mRNA was observed (Figure 1A). Similarly, LMP1 increased the levels of both precursor and mature forms of SREBP1 protein (Figure 1C). SREBP1 transactivates target genes by binding to SREs within the promoter region. Using a pGL2‐3xSRE reporter construct containing three tandem copies of an SRE/SP1 element 18, we found that LMP1 increased *SREBP1* transcriptional activity (Figure 1B). FASN is a transcriptional target of SREBP1. FASN promoter activity was also strongly enhanced in response to LMP1 expression (Figure 1B). The expression of *FASN* mRNA and the expression of FASN protein were also induced by LMP1 (Figure 1A,C). These findings suggest that FASN induction by LMP1 is mediated primarily through modulation of the expression and activity of SREBP1. The mTORC1 and mTORC2 pathways are both involved in regulating SREBP1 activity 12, 13, 14. LMP1 has been reported to activate the mTOR signaling pathway 18, 19, 20. Here, we provided further evidence that LMP1 induces phosphorylation of 4E‐BP1 and p70S6K, two established downstream targets of mTOR signaling (Figure 1C).

In nasopharyngeal epithelial cells stably expressing LMP1, we also observed increased activities of the SREBP1 and mTOR pathways (Figure 1D). Immunofluorescence staining revealed overexpression of FASN in LMP1‐expressing nasopharyngeal epithelial cells (Figure 1E). To determine whether LMP1 promotes *de novo* lipogenesis, LMP1‐expressing cells were labeled with [^14^C]acetate for 8–12 h under serum‐deprived conditions. The ^14^C‐labeled lipid fraction was then extracted for quantification. As shown in Figure 1F, LMP1‐expressing cells produced more newly synthesized lipid than control cells. Furthermore, Nile Red staining revealed more intracellular lipid droplets in LMP1‐expressing cells (Figure 1G,H). Overall, these data indicate a role for LMP1 in promoting lipid biosynthesis.

### LMP1 promotes SREBP1‐mediated lipogenesis in EBV‐infected NPC cells

**Figure 2:**
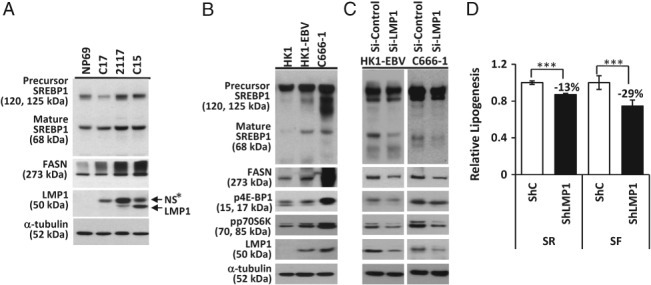
LMP1 induction of SREBP1‐mediated lipogenesis in EBV‐infected NPC cells. (A) NPC xenografts (C17, 2117, and C15) together with cells of the immortalized nasopharyngeal epithelial cell line, NP69, which were incubated in serum‐free medium for 16 h prior to harvesting, were subjected to western blotting analysis for the indicated proteins. (B) The EBV‐infected NPC cell lines HK1‐EBV and C666‐1, together with EBV‐negative HK‐1 cells, were incubated in serum‐free medium for 12 h prior to western blotting analysis. (C) HK1‐EBV and C666‐1 cells transfected with negative siRNA control (si‐Control) or siRNA targeting LMP1 (si‐LMP1) were subjected to western blotting analysis. (D) C666‐1 cells expressing pSuper.retro‐shRNA scrambled control (ShC) or shRNA LMP1 (ShLMP1) were incubated in serum‐rich (SR) or serum‐free (SF) medium containing 2.5 μCi/ml [1‐^14^C]acetate for 10 h, and then subjected to measurement of lipid synthesis. NS*, non‐specific band. Mean and standard deviation. ***p < 0.001.

In NPC xenografts (C17, X2117, and C15), western blotting analysis revealed higher levels of the precursor and mature forms of SREBP1 and FASN in the LMP1‐positive X2117 and C15 xenografts than in the LMP1‐negative C17 xenograft and EBV‐negative non‐malignant NP69 nasopharyngeal epithelial cell line (Figure 2A). In NPC cell lines, the levels of mature SREBP1 and FASN proteins were higher in EBV‐infected HK1‐EBV and C666‐1 cells than in HK‐1 cells (Figure 2B). Similarly, levels of the phosphorylated forms of 4E‐BP1 and p70S6K were elevated in HK1‐EBV and C666‐1 cells (Figure 2B). A role for LMP1 in these effects was established, as siRNA silencing of LMP1 in EBV‐infected cells resulted in reductions in the expression levels of mature SREBP1 and FASN, as well as a reduction in the signaling activity of mTOR (Figure 2C). Furthermore, LMP1 silencing in C666‐1 cells resulted in a decrease in FASN promoter activity (supplementary material, Figure S1) as well as lipogenesis, particularly under serum‐deprived condition (Figure 2D). These data suggest that SREPB1‐mediated lipogenesis in EBV‐infected cells is induced by LMP1 through the mTOR signaling pathway.

### LMP1 induces SREBP1‐mediated lipogenesis through the mTOR signaling pathway

**Figure 3:**
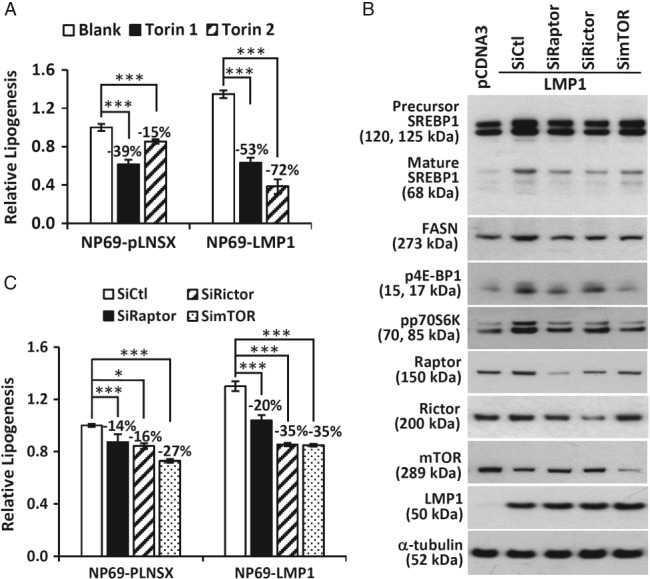
LMP1 activation of the mTOR signaling pathway is required for SREBP1‐mediated lipogenesis. (A) NP69‐pLNSX and NP69‐LMP1 cells treated with vehicle alone (Blank), Torin 1 (0.3 μm) or Torin 2 (0.1 μm). (B) NP69 cells transfected with either control pCDNA3 vector or pCDNA3‐LMP1 expression vector, together with a control scrambled siRNA (siCtl), or siRNA specific for Raptor (siRaptor), Rictor (siRictor), or mTOR (simTOR), were subjected to western blotting analysis for the indicated proteins. (C) NP69‐pLNSX and NP69‐LMP1 cells transfected with the indicated siRNAs for 24 h were subjected to lipid synthesis measurements. The asterisks indicate a significant difference (*p < 0.05, **p < 0.01, ***p < 0.001).

To investigate whether LMP1‐induced lipogenesis was dependent on mTOR signaling, NP69‐pLNSX and NP69‐LMP1 cells were treated with the mTOR inhibitors Torin 1 and Torin 2. Torin 1 has been shown to inhibit both mTORC1 and mTORC2 complexes, whereas Torin 2 inhibits the mTORC1 complex 21, 22. [^14^C]Acetate incorporation assays revealed that these two mTOR inhibitors could suppress lipogenesis in both cell lines, although the effects were more profound in LMP1‐expressing cells. As shown in Figure 3A, NP69‐LMP1 cells showed a 53–72% reduction in lipogenesis, whereas only a 15–39% reduction was observed in NP69‐pLNSX cells. To further investigate whether LMP1 induction of mTOR signaling promoted SREBP1‐mediated lipogenesis, NP69 cells were transiently transfected with an LMP1 expression vector together with siRNAs targeting raptor to inhibit mTORC1 signaling, rictor to inhibit mTORC2 signaling, or mTOR to inhibit both mTORC1 and mTORC2 signaling. As shown in Figure 3B, inhibition of either mTORC1, mTORC2 or both reduced LMP1‐induced FASN expression and SREBP1 maturation. Also, NP69‐LMP1 cells showed a 20–35% reduction in lipogenesis, whereas a 14–27% reduction was observed in control NP69‐pLNSX cells (Figure 3C). These findings indicated that mTOR signaling contributes to LMP1‐induced lipogenesis.

### Expression of FASN correlates with LMP1 expression in NPCs and poor prognosis in NPC patients

**Figure 4:**
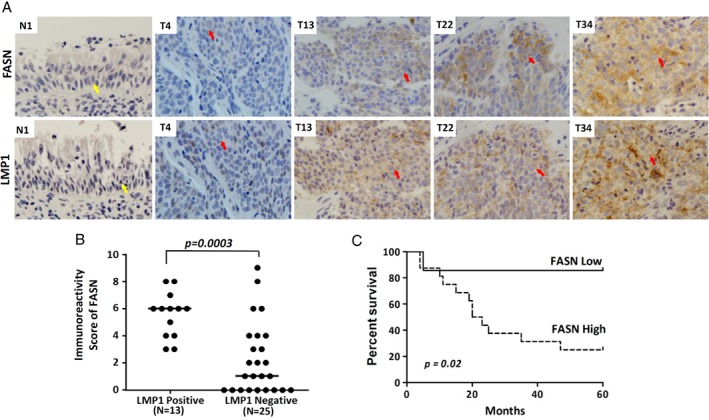
Elevation of FASN expression correlates with LMP1 expression in NPC tumors and is associated with poor prognosis in NPC patients. (A) Immunohistochemical staining of FASN and LMP1 in primary NPC and normal nasopharyngeal epithelium (NE) specimens. An absence of FASN and LMP1 expression was observed in NE (N1). In NPC tumor T4, which is LMP1‐negative, low levels of FASN (immunoreactivity score of 1) were observed. In NPC tumors T13, T22, and T34, which are LMP1‐positive, moderate (immunoreactivity score of 4) and high (immunoreactivity scores of 6 and 8) levels of FASN were observed. Yellow arrow: normal nasopharyngeal epithelium. Red arrow: NPC tumor cells. (B) Dot plot showing the immunoreactivity scores of FASN staining within the group of tumors with and without LMP1 expression (*N* = 38). All LMP1‐positive NPC tumors (13/13) showed strong FASN expression (immunoreactivity score of ≥3). The median value of each group is shown by the horizontal line. The *p*‐value between two groups is shown. (C) Kaplan–Meier survival curves for NPC patients with available follow‐up information. High FASN expression: immunoreactivity score of ≥3, *n* = 16. Low FASN expression: immunoreactivity score of <3, *n* = 7.

**Figure 5:**
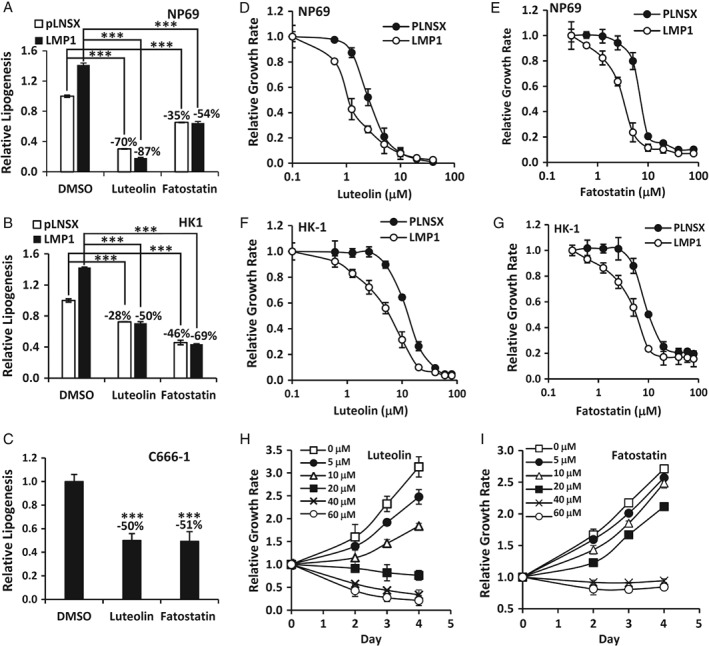
LMP1 induction of lipogenesis contributes to cell proliferation. (A–C) NP69‐pLNSX and NP69‐LMP1 cells (A), HK1‐pLNSX and HK1‐LMP1 cells (B) and C6661‐1 EBV‐positive NPC cells (C) cultured with 1% serum were treated with dimethylsulfoxide (DMSO) (vehicle), luteolin (20 μm) or fatostatin (15 μm) for 16 h prior to lipogenesis measurements. Relative lipogenesis was calculated with vehicle‐treated cells as the reference control (set at 1). (D–I) NP69‐pLNSX and NP69‐LMP1 cells (D and E), HK1‐pLNSX and HK1‐LMP1 cells (F and G) and C666‐1 cells (H and I) were treated with increasing doses of inhibitors, as indicated, for 3 days prior to cell growth analysis. Relative cell growth was calculated with vehicle‐treated cells as the reference control (set at 1). ***p < 0.001.

To examine the expression of FASN in NPC, we performed immunohistochemical staining for FASN and LMP1 in 38 NPC primary tumors. The intensity of FASN staining was scored, and a graph of the statistical dot‐plot of FASN staining intensity against LMP1 expression was generated (Figure 4B). Immunohistochemical staining revealed absent or low expression of FASN (immunoreactivity score of <3) in normal nasopharyngeal epithelium (Figure 4A, N1), and 16 of 38 (42%) NPC tumors in which LMP1 expression was barely detectable (Figure 4A, representative NPC: T4). In contrast, moderate or high levels of FASN (immunoreactivity score of ≥3) were observed in 22 of 38 NPC tumors (58%) (Figure 5A, representative NPC: T13, T22, and T34). In particular, LMP1‐positive tumors showed significantly higher levels of FASN (*p* = 0.0003) (Figure 4B). In analysis of FASN expression with clinicopathological variables including gender, age, tumor size, lymph node and cancer stage in NPC patients, we found that higher levels of FASN expression (score of ≥3) significantly correlated with advanced primary tumor (T3–T4; *p* = 0.012) and distant lymph node metastasis (N1–N3; *p* = 0.005) of NPC (supplementary material, Table S1). Also, Kaplan–Meier survival analysis revealed that elevated FASN expression significantly correlated with poor survival (*p* = 0.02) (Figure 4C). Overall, these findings indicate that FASN overexpression is common in NPC and is correlated with LMP1 expression. Moreover, the elevated FASN expression is associated with aggressive disease and poor prognosis in NPC patients. These also imply a role of LMP1 in upregulating FASN for NPC progression.

### LMP1 induction of lipid synthesis contributes to cell growth

Next, we examined whether LMP1 induction of lipogenesis contributed to cell growth. NP69 and HK1 cells expressing pLNSX control or LMP1 were treated with luteolin or fatostatin. Luteolin is an inhibitor that suppresses SREBP‐mediated lipogenesis. Fatostatin is an SREBP‐specific inhibitor 23, 24, 25. [^14^C]Acetate incorporation assays revealed that these inhibitors could dampen lipogenesis in all cell lines; however, the repression was more evident in LMP1‐expressing cells (Figure 5A,B). Fatostatin and luteolin treatment reduced lipogenesis of NP69‐pLNSX cells by 35–70%, whereas a 54–87% reduction was observed in NP69‐LMP1 cells (Figure 5A). Similarly, fatostatin and luteolin reduced lipogenesis in HK1‐pLNSX cells by 28–46% and in HK1‐LMP1 cells by 50–69% (Figure 5B). Western blot analysis confirmed that both luteolin and fatostatin effectively inhibited SREBP1 maturation and FASN expression in NP69‐LMP1 and HK1‐LMP1 cells (supplementary material, Figure S2).

To determine whether lipogenesis conferred a growth advantage to LMP1‐expressing cells, pLNSX control and LMP1‐expressing NP69 and HK‐1 cells were grown in culture medium supplemented with 1% serum together with increasing doses of luteolin or fatostatin for 1–4 days, and the effects on cell growth were examined. As shown in Figure 5D–G, inhibition of fatty acid synthesis by luteolin or fatostatin reduced the proliferation of both control and LMP1‐expressing cells, although NP69‐LMP1 and HK1‐LMP1 cells appeared to be more susceptible to the actions of both drugs. The IC_50_ values of luteolin at day 3 in NP69‐pLNSX, NP69‐LMP1, HK1‐pLNSX and HK1‐LMP1 cells were 2.6, 1.14, 12.4 and 5.6 μm, respectively. Similarly, the IC_50_ values of fatostatin at day 3 in NP69‐pLNSX, NP69‐LMP1, HK1‐pLNSX and HK1‐LMP1 cells were 6.5, 2.9, 5.6 and 3.3 μm, respectively. Also, these two inhibitors suppressed lipogenesis in C666‐1 NPC cells by 50% (Figure 5C) and inhibited their proliferation; the IC_50_ values of luteolin and fatostatin at day 4 were 9.6 and 25.5 μm, respectively (Figure 5H,I). These findings indicate that suppression of lipogenesis inhibits LMP1‐induced proliferation in both malignant and non‐malignant nasopharyngeal epithelial cells, implying that the induction of lipogenesis by LMP1 is an essential mechanism in facilitating cell proliferation.

### Inhibitors of lipogenesis suppress NPC growth and induce apoptosis *in vivo*

**Figure 6:**
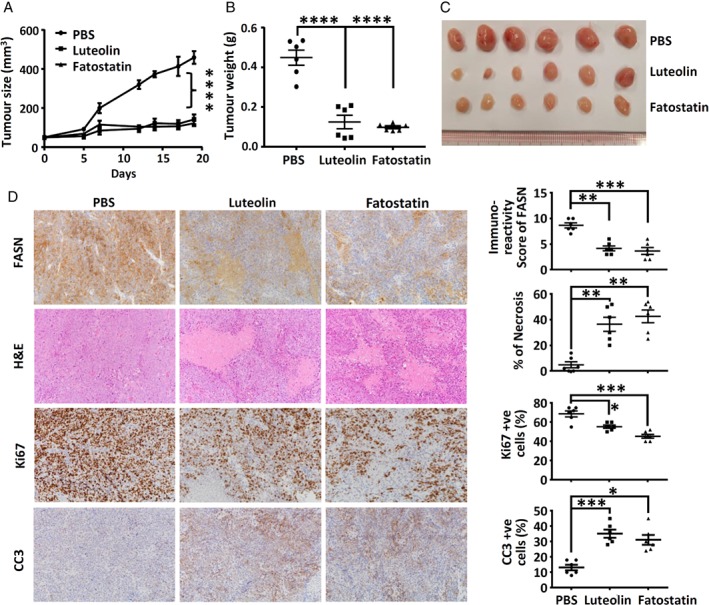
Suppression of NPC tumor growth by inhibitors of lipogenesis. Mice bearing C666‐1 xenografts were treated with PBS (control), luteolin (20 mg/kg) or fatostatin (15 mg/kg) every 2–3 days for 3 weeks. (A) Tumor size was measured every 2–3 days for 19 days. (B and C) At the endpoint, mice were killed, and tumors were (B) weighed and (C) photographed. (D, left panel) The harvested xenografts were embedded for hematoxylin and eosin (H&E) staining and immunohistochemical staining analysis. (D, right panel) The percentage of necrosis, and positive staining for FASN, Ki67, and CC3, were measured, and are shown as dot‐plots. Lines and bars are mean and standard error of the mean of each experimental group. *p < 0.05, **p < 0.01, ***p < 0.001, ****p < 0.0001.

Next, we examined whether blocking lipogenesis inhibited the growth of NPC tumors *in vivo*. C666‐1 cells were subcutaneously injected into the flanks of nude mice. When tumors reached 50 mm^3^ in size, the mice were injected intraperitoneally with PBS, luteolin (20 mg/kg) or fatostatin (15 mg/kg) every 2–3 days for 19 days. Body weight and tumor size were measured every several days. At the endpoint, mice were killed and tumors were harvested. Both luteolin and fatostatin significantly inhibited tumor growth in mice (*p* < 0.0001) (Figure 6A). In mice treated with luteolin or fatostatin, substantially lower tumor weights and smaller tumor sizes were observed (*p* < 0.0001) (Figure 6B,C). Surprisingly, no noticeable weight loss was observed in mice treated with either drug (supplementary material, Figure S3). Immunohistochemical staining analysis of NPC tumors from these two groups of mice indicated that both luteolin and fatostatin effectively inhibited FASN expression (Figure 6D; supplementary material, Figure S4). In addition, histological investigations of tumor tissues revealed more pronounced necrosis in both the luteolin‐treated (35%) and fatostatin‐treated (43%) groups than in the control (5%) group (Figure 6D). An examination of cell proliferation by immunohistochemical staining for Ki67, and apoptosis by staining for cleaved caspase‐3 (CC3), revealed that both drugs significantly inhibited cell proliferation and increased apoptosis. As shown in Figure 6D, the percentages of Ki67‐positive cells were markedly lower in the tumor tissues of the luteolin‐treated (56%) and fatostatin‐treatyed (45%) groups than in the control group (70%). Also, the percentages of CC3‐positive cells were significantly higher in the luteolin‐treated (36%) and fatostatin‐treated (31%) treated groups than in the control group (12%). Western blotting analysis provided further evidence for the induction of CC3 and cleaved poly(ADP‐ribose) polymerase in luteolin‐treated and fatostatin‐treated tumors (supplementary material, Figure S4). These findings indicate that inhibition of lipogenesis by luteolin and fatostatin causes cell apoptosis and necrosis, leading to tumor growth suppression.

## Discussion

The oncogenic LMP1 protein is frequently found to be expressed in NPC tumors. As well as promoting cell growth, transformation, invasion, and migration, LMP1 also plays a role in modulating host cell metabolic pathways 1, 3, 26. In previous studies, we have demonstrated an ability of LMP1 to promote aerobic glycolysis through constitutive activation of the fibroblast growth factor 2–fibroblast growth factor receptor 1 signaling pathway and upregulation of c‐Myc and hypoxia‐inducible factor (HIF) 1α, which are two primary regulators of aerobic glycolysis 5. LMP1 also inactivates liver kinase B1 (LKB1)–5′‐AMP‐activated protein kinase (AMPK) to promote proliferation and anchorage‐independent growth 4. Here, we report a novel function of LMP1 in promoting SREBP1‐mediated *de novo* lipogenesis, an effect that facilitates cell growth and tumor development (supplementary material, Figure S5).

In this study, we show that LMP1 increases SREBP1 expression and activity in nasopharyngeal epithelial cells. Luciferase promoter reporter assays and RT‐qPCR analysis demonstrate that LMP1 upregulates SREBP1 at the transcriptional level (Figure 1A,B), and western blotting analysis demonstrates that LMP1 promotes SREBP1 maturation and the expression of its downstream target FASN (Figure 1C,D). Using a [^14^C]acetate incorporation assay, we demonstrate the ability of LMP1 to promote lipogenesis (Figure 1F). Interestingly, silencing LMP1 in EBV‐infected NPC cells resulted in a reduction in FASN expression and lipogenesis (Figure 2C,D). Furthermore, suppressing lipogenesis with inhibitors significantly reduced LMP1‐induced proliferation (Figure 5). Collectively, these findings demonstrate that LMP1 induction of lipogenesis in NPC contributes to cell proliferation.

SREBPs tightly regulate lipogenesis. However, the mechanism underlying the expression and activity of SREBPs is still unclear. Hypoxia, glucose and insulin have been found to increase the expression of SREBPs 10. Interestingly, we and others have reported a function of LMP1 in promoting HIF‐1 expression and glucose uptake 5, 26. mTORC1 and mTORC2 have been shown to regulate SREBP activity and lipogenesis through multiple inputs 27, 28, 29, 30. Here, in line with other studies 18, 19, 20, we demonstrate that LMP1 increases the phosphorylation of 4E‐BP1 and p70S6K, which are two established targets of mTOR, in nasopharyngeal epithelial cells (Figures 1, 2, 3). Using siRNA targeting raptor or rictor, we show that both the mTORC1 and mTORC2 signaling pathways are involved in LMP1‐mediated lipogenesis (Figure 3). The mTOR signaling pathway is complicated, as it contains both positive and negative feedback loops, and its downstream signaling interconnects with other cell growth and survival pathways 28, 29, 30, 31. mTORC2 is activated by ribosomes and PI3K signaling, and is negatively regulated by an mTORC1‐induced p70S6 kinase, which dampens PI3K signaling by inhibiting insulin receptor substrate 1 28, 29, 30, 31. mTORC1 activity is induced by the PI3K–AKT, Ras–ERK–MAPK, IKKα–nuclear factor‐αB (NF‐αB) and IKKβ–NF‐αB pathways, all of which inhibit tuberous sclerosis complex (TSC), a critical negative regulator of mTORC1. mTORC2‐induced AKT signaling also activates mTORC1. In contrast, LKB1–AMPK inhibits mTORC1 by activating TSC2 and inactivating raptor 28, 29, 30, 31. Interestingly, AMPK is an inhibitory target of LMP1 4. PI3K–AKT, Ras–ERK–MAPK and NF‐κB are three major pathways engaged by LMP1 [2;3]. Thus, LMP1 induction of mTOR–SREBP‐induced lipogenesis appears to be mediated through multiple downstream targets and/or signaling pathways.

Overexpression of FASN has been documented in oral hairy leukoplakia, an EBV‐associated benign lesion associated with robust EBV lytic replication 32. FASN is induced by the EBV‐encoded lytic protein BRLF‐1 through the p38‐MAPK pathway, and is required for lytic viral gene expression 32. Similarly, hepatitis B virus and hepatitis C virus lytic infection in liver cancer cells is associated with the induction of fatty acid synthesis for the formation of viral envelopes 33, 34, 35. Another γ‐herpesvirus, Kaposi's sarcoma‐associated herpesvirus, has been shown to induce FASN expression and fatty acid synthesis for the survival of latently infected PEL cells 36. Here, we found higher levels of SREBP1 activation and FASN expression in EBV latently infected NPC cells and xenografts, in which LMP1 was expressed (Figure 2). Whereas normal nasopharyngeal epithelium showed an absence of expression of FASN and LMP1 (Figure 4A), a high level of FASN expression in NPC primary tumors was common, and correlated significantly with LMP1 expression (Figure 4A,B). In line with previous studies, elevated FASN expression was significantly associated with T3–T4 stage primary tumors, N1–N3 lymph node metastasis and poor overall survival of NPC patients (Figure 4C; supplementary material, Table S1) 37. These findings suggest that LMP1 induction of FASN and lipogenesis is involved in NPC progression. Among the LMP1‐negative NPC tumors examined, nine of 25 (36%) showed a moderate or high level of FASN expression (Figure 4B). Our previous genetic analyses indicated that somatic mutations and/or aberrant expression of signaling proteins (lymphotoxin β receptor, PIK3CA, p50, RelB, Bcl3, epidermal growth factor receptor, and RAS) or signaling regulators (INPP4B, TRAF3, TRAF2, A20, NFKBIA, TNFAIP3, and CYLD) are common in NPC, resulting in constitutive activation of the ERK1/2, NF‐κB and PI3K–AKT pathways 38, 39, 40. Therefore, the genetic background of NPC tumors, in addition to LMP1 expression, is likely to contribute to FASN upregulation.

Given that FASN expression is commonly increased in virus‐associated cancers, lipogenesis appears to be essential for viral infection and cancer progression 35. As lipogenesis is not common in normal cells, targeting lipogenesis (or lipogenic pathways) might selectively inhibit the growth of virus‐infected cells and of highly proliferative cancer cells in the early stage of cancer development. In this study, we examined the effects of fatostatin and luteolin on cell growth and tumor development in relation to NPC. Fatostatin is a small molecule that specifically blocks proteolytic activation of SREBPs 24. Luteolin is a plant flavonoid that has inhibitory effects on lipogenesis 23. Both fatostatin and luteolin significantly inhibit SREBP1 activity, lipid synthesis and cell proliferation induced by LMP1 (Figure 5; supplementary material, Figure S2). Interestingly, luteolin has been reported to inhibit the signaling activities of the PI3K–AKT, MAPK–ERK and mTOR pathways 41, which are the major pathways required for SREBP‐mediated lipogenesis by LMP1. Moreover, fatostatin and luteolin significantly inhibit the proliferation and tumorigenic growth of C666‐1 cells. In nude mice, fatostatin and luteolin at a doses that effectively downregulate FASN expression caused a marked reduction in tumor growth (Figure 6; supplementary material, Figure S4). Surprisingly, no significant weight loss was observed in mice treated with luteolin or fatostatin (supplementary material, Figure S3). The promising effects of these inhibitors in suppressing NPC tumor growth with low‐level toxicity suggest possible clinical utility for the treatment of NPC. Luteolin is a dietary flavonoid found in vegetables, fruits, and herbs. In addition to its inhibitory effect on lipogenesis, luteolin suppresses inflammation, angiogenesis, cell proliferation, and metastasis, all of which are associated with cancer development 41. Interestingly, luteolin has been shown to induce G_1_ arrest and inhibit EBV reactivation in NPC cells 42, 43. Given that luteolin is a natural polyphenolic compound with low toxicity, it may prove to be a promising compound for the inhibition of EBV infection and the treatment of EBV‐associated cancers. The potential of luteolin for the treatment of EBV‐associated malignancies is worthy of further evaluation, particularly in clinical trials.

## Author contributions statement

AKFL, RWML, CWD, LSY and KWL designed research. AKFL, RWML, CWD, CWK, WWY, WK, KFT and KWL performed research. AKFL, RWML, CWD, LSY and KWL analyzed data and drafted the manuscript. All authors discussed the findings, reviewed the data, and commented on the manuscript.

**SUPPLEMENTARY MATERIAL ONLINE**

**Supplementary materials and methods**

**Figure S1.** Silencing of LMP1 leads to a reduction of FASN promoter activity

**Figure S2.** The inhibitory effects of luteolin and fatostatin on SREBP1 maturation and FASN expression

**Figure S3.** Body weight of mice bearing C666‐1 NPC xenografts during the course of treatment with either PBS (control), luteolin, or fatostatin

**Figure S4.** The inhibitory effects of Luteolin and Fatostatin on FASN expression, cell proliferation and apoptosis in NPC xenografts

**Figure S5.** The mechanism of LMP1 in upregulation of lipogenesis

**Table S1.** Clinicopathologic characteristics of patients with NPC

## Supporting information


**Supplementary materials and methods**

**Figure S1. Silencing of LMP1 leads to a reduction of** *FASN* **promoter activity.** C666‐1 were transfected with either a negative control siRNAs (SiCtl) or LMP1 siRNA (siLMP1) together with the pGL3‐FASN luciferase promoter vector. After incubation in serum‐free medium for 12 h, cells were harvested for luciferase analysis. Luciferase activity was normalized to Renilla activity and was plotted relative to the siRNA control (SiCtl) (set at 1).
**Figure S2. The inhibitory effects of Luteolin and Fatostatin on SREBP1 maturation and FASN expression.** Western blotting analysis of SREBP1 and FASN in LMP1 expressing NP69 and HK‐1 nasopharyngeal epithelial cells treated with luteolin or fatostatin.
**Figure S3**. Body weight of mice bearing C666‐1 NPC xenografts during the course (19 days) of treatment with PBS (control), luteolin (20 mg/kg), fatostatin (20 mg/kg).
**Figure S4. The inhibitory effects of Luteolin and Fatostatin on FASN expression, cell proliferation and apoptosis in NPC xenografts.** Western blotting analysis of FASN, cleaved PARP and Cleaved Caspase 3 proteins in NPC tumours harvested from animals treated with PBS, (A) luteolin or (B) fatostatin.
**Figure S5. The mechanism of LMP1 in upregulation of lipogenesis.** Induction of mTOR by LMP 1 increases/activates SREBP1‐meditated lipogenesis, facilitating cell proliferation and NPC tumour growth.

**Table S1**. Clinicopathologic characteristics of patients with NPC

## References

[1] Tsao SW , Tsang CM , To KF , *et al* The role of Epstein–Barr virus in epithelial malignancies. J Pathol 2015; 235 **:** 323–333. 25251730 10.1002/path.4448 PMC4280676

[2] Young LS , Dawson CW . Epstein–Barr virus and nasopharyngeal carcinoma. Chin J Cancer 2014; 33 **:** 581–590. 25418193 10.5732/cjc.014.10197 PMC4308653

[3] Dawson CW , Port RJ , Young LS . The role of the EBV‐encoded latent membrane proteins LMP1 and LMP2 in the pathogenesis of nasopharyngeal carcinoma (NPC). Semin Cancer Biol 2012; 22 **:** 144–153. 22249143 10.1016/j.semcancer.2012.01.004

[4] Lo AK , Lo KW , Ko CW , *et al* Inhibition of the LKB1–AMPK pathway by the Epstein–Barr virus‐encoded LMP1 promotes proliferation and transformation of human nasopharyngeal epithelial cells. J Pathol 2013; 230 **:** 336–346. 23592276 10.1002/path.4201

[5] Lo AK , Dawson CW , Young LS , *et al* Activation of the FGFR1 signalling pathway by the Epstein–Barr virus‐encoded LMP1 promotes aerobic glycolysis and transformation of human nasopharyngeal epithelial cells. J Pathol 2015; 237 **:** 238–248. 26096068 10.1002/path.4575

[6] Baenke F , Peck B , Miess H , *et al* Hooked on fat: the role of lipid synthesis in cancer metabolism and tumour development. Dis Model Mech 2013; 6 **:** 1353–1363. 24203995 10.1242/dmm.011338 PMC3820259

[7] Beloribi‐Djefaflia S , Vasseur S , Guillaumond F . Lipid metabolic reprogramming in cancer cells. Oncogenesis 2016; 5 **:** e189. 10.1038/oncsis.2015.49 PMC4728678 26807644

[8] Currie E , Schulze A , Zechner R , *et al* Cellular fatty acid metabolism and cancer. Cell Metab 2013; 18 **:** 153–161. 23791484 10.1016/j.cmet.2013.05.017 PMC3742569

[9] Guo D , Bell EH , Mischel P , *et al* Targeting SREBP‐1‐driven lipid metabolism to treat cancer. Curr Pharm Des 2014; 20 **:** 2619–2626. 23859617 10.2174/13816128113199990486 PMC4148912

[10] Rohrig F , Schulze A . The multifaceted roles of fatty acid synthesis in cancer. Nat Rev Cancer 2016; 16 **:** 732–749. 27658529 10.1038/nrc.2016.89

[11] Shao W , Espenshade PJ . Expanding roles for SREBP in metabolism. Cell Metab 2012; 16 **:** 414–419. 23000402 10.1016/j.cmet.2012.09.002 PMC3466394

[12] Ricoult SJ , Manning BD . The multifaceted role of mTORC1 in the control of lipid metabolism. EMBO Rep 2013; 14 **:** 242–251. 23399656 10.1038/embor.2013.5 PMC3589096

[13] Shimano H , Sato R . SREBP‐regulated lipid metabolism: convergent physiology – divergent pathophysiology. Nat Rev Endocrinol 2017; 13 **:** 710–730. 28849786 10.1038/nrendo.2017.91

[14] Ricoult SJ , Yecies JL , Ben‐Sahra I , *et al* Oncogenic PI3K and K‐Ras stimulate de novo lipid synthesis through mTORC1 and SREBP. Oncogene 2016; 35 **:** 1250–1260. 26028026 10.1038/onc.2015.179 PMC4666838

[15] Jackel‐Cram C , Babiuk LA , Liu Q . Up‐regulation of fatty acid synthase promoter by hepatitis C virus core protein: genotype‐3a core has a stronger effect than genotype‐1b core. J Hepatol 2007; 46 **:** 999–1008. 17188392 10.1016/j.jhep.2006.10.019

[16] Lo AK , Lo KW , Tsao SW , *et al* Epstein–Barr virus infection alters cellular signal cascades in human nasopharyngeal epithelial cells. Neoplasia 2006; 8 **:** 173–180. 16611410 10.1593/neo.05625 PMC1578522

[17] Lo AK , To KF , Lo KW , *et al* Modulation of LMP1 protein expression by EBV‐encoded microRNAs. Proc Natl Acad Sci U S A 2007; 104 **:** 16164–16169. 17911266 10.1073/pnas.0702896104 PMC2042179

[18] Hua X , Nohturfft A , Goldstein JL , *et al* Sterol resistance in CHO cells traced to point mutation in SREBP cleavage‐activating protein. Cell 1996; 87 **:** 415–426. 8898195 10.1016/s0092-8674(00)81362-8

[19] Zhang J , Jia L , Lin W , *et al* Epstein–Barr virus‐encoded latent membrane protein 1 upregulates glucose transporter 1 transcription via the mTORC1/NF‐kappaB signaling pathways. J Virol 2017; 91: e02168–16. 10.1128/JVI.02168-16 PMC5331802 28053105

[20] Lambert SL , Martinez OM . Latent membrane protein 1 of EBV activates phosphatidylinositol 3‐kinase to induce production of IL‐10. J Immunol 2007; 179 **:** 8225–8234. 18056366 10.4049/jimmunol.179.12.8225

[21] Thoreen CC , Kang SA , Chang JW , *et al* An ATP‐competitive mammalian target of rapamycin inhibitor reveals rapamycin‐resistant functions of mTORC1. J Biol Chem 2009; 284 **:** 8023–8032. 19150980 10.1074/jbc.M900301200 PMC2658096

[22] Liu Q , Xu C , Kirubakaran S , *et al* Characterization of Torin2, an ATP‐competitive inhibitor of mTOR, ATM, and ATR. Cancer Res 2013; 73 **:** 2574–2586. 23436801 10.1158/0008-5472.CAN-12-1702 PMC3760004

[23] Brusselmans K , Vrolix R , Verhoeven G , *et al* Induction of cancer cell apoptosis by flavonoids is associated with their ability to inhibit fatty acid synthase activity. J Biol Chem 2005; 280 **:** 5636–5645. 15533929 10.1074/jbc.M408177200

[24] Kamisuki S , Mao Q , Abu‐Elheiga L , *et al* A small molecule that blocks fat synthesis by inhibiting the activation of SREBP. Chem Biol 2009; 16 **:** 882–892. 19716478 10.1016/j.chembiol.2009.07.007

[25] Kuhajda FP , Pizer ES , Li JN , *et al* Synthesis and antitumor activity of an inhibitor of fatty acid synthase. Proc Natl Acad Sci U S A 2000; 97 **:** 3450–3454. 10716717 10.1073/pnas.050582897 PMC16260

[26] Lo AK , Dawson CW , Young LS , *et al* The role of metabolic reprogramming in gamma‐herpesvirus‐associated oncogenesis. Int J Cancer 2017; 141 **:** 1512–1521. 28542909 10.1002/ijc.30795

[27] Ilagan E , Manning BD . Emerging role of mTOR in the response to cancer therapeutics. Trends Cancer 2016; 2 **:** 241–251. 27668290 10.1016/j.trecan.2016.03.008 PMC5033243

[28] Saxton RA , Sabatini DM . mTOR signaling in growth, metabolism, and disease. Cell 2017; 168 **:** 960–976. 28283069 10.1016/j.cell.2017.02.004 PMC5394987

[29] Guri Y , Hall MN . mTOR signaling confers resistance to targeted cancer drugs. Trends Cancer 2016; 2 **:** 688–697. 28741507 10.1016/j.trecan.2016.10.006

[30] Ben‐Sahra I , Manning BD . mTORC1 signaling and the metabolic control of cell growth. Curr Opin Cell Biol 2017; 45 **:** 72–82. 28411448 10.1016/j.ceb.2017.02.012 PMC5545101

[31] Dan HC , Adli M , Baldwin AS . Regulation of mammalian target of rapamycin activity in PTEN‐inactive prostate cancer cells by I kappa B kinase alpha. Cancer Res 2007; 67 **:** 6263–6269. 17616684 10.1158/0008-5472.CAN-07-1232

[32] Li Y , Webster‐Cyriaque J , Tomlinson CC , *et al* Fatty acid synthase expression is induced by the Epstein–Barr virus immediate‐early protein BRLF1 and is required for lytic viral gene expression. J Virol 2004; 78 **:** 4197–4206. 15047835 10.1128/JVI.78.8.4197-4206.2004 PMC374282

[33] Okamura H , Nio Y , Akahori Y , *et al* Fatty acid biosynthesis is involved in the production of hepatitis B virus particles. Biochem Biophys Res Commun 2016; 475 **:** 87–92. 27178211 10.1016/j.bbrc.2016.05.043

[34] Syed GH , Amako Y , Siddiqui A . Hepatitis C virus hijacks host lipid metabolism. Trends Endocrinol Metab 2010; 21 **:** 33–40. 19854061 10.1016/j.tem.2009.07.005 PMC2818172

[35] Ameer F , Scandiuzzi L , Hasnain S , *et al* De novo lipogenesis in health and disease. Metabolism 2014; 63 **:** 895–902. 24814684 10.1016/j.metabol.2014.04.003

[36] Bhatt AP , Jacobs SR , Freemerman AJ , *et al* Dysregulation of fatty acid synthesis and glycolysis in non‐Hodgkin lymphoma. Proc Natl Acad Sci U S A 2012; 109 **:** 11818–11823. 22752304 10.1073/pnas.1205995109 PMC3406848

[37] Kao YC , Lee SW , Lin LC , *et al* Fatty acid synthase overexpression confers an independent prognosticator and associates with radiation resistance in nasopharyngeal carcinoma. Tumour Biol 2013; 34 **:** 759–768. 23208675 10.1007/s13277-012-0605-y

[38] Chung GT , Lou WP , Chow C , *et al* Constitutive activation of distinct NF‐kappaB signals in EBV‐associated nasopharyngeal carcinoma. J Pathol 2013; 231 **:** 311–322. 23868181 10.1002/path.4239

[39] Li YY , Chung GT , Lui VW , *et al* Exome and genome sequencing of nasopharynx cancer identifies NF‐kappaB pathway activating mutations. Nat Commun 2017; 8 **:** 14121. 28098136 10.1038/ncomms14121 PMC5253631

[40] Yuen JW , Chung GT , Lun SW , *et al* Epigenetic inactivation of inositol polyphosphate 4‐phosphatase B (INPP4B), a regulator of PI3K/AKT signaling pathway in EBV‐associated nasopharyngeal carcinoma. PLoS One 2014; 9 **:** e105163. 10.1371/journal.pone.0105163 PMC4134277 25126743

[41] Seelinger G , Merfort I , Wolfle U , *et al* Anti‐carcinogenic effects of the flavonoid luteolin. Molecules 2008; 13 **:** 2628–2651. 18946424 10.3390/molecules13102628 PMC6245397

[42] Wu CC , Fang CY , Hsu HY , *et al* EBV reactivation as a target of luteolin to repress NPC tumorigenesis. Oncotarget 2016; 7 **:** 18999–19017. 26967558 10.18632/oncotarget.7967 PMC4951347

[43] Ong CS , Zhou J , Ong CN , *et al* Luteolin induces G1 arrest in human nasopharyngeal carcinoma cells via the Akt‐GSK‐3beta‐Cyclin D1 pathway. Cancer Lett 2010; 298 **:** 167–175. 20655656 10.1016/j.canlet.2010.07.001

