# Supplementary material for: Activation of sterol regulatory element‐binding protein 1 (SREBP1)‐mediated lipogenesis by the Epstein–Barr virus‐encoded latent membrane protein 1 (LMP1) promotes cell proliferation and progression of nasopharyngeal carcinoma
Source: J Pathol. 2018 Aug 22;246(2):180–90. doi: 10.1002/path.5130 (PMC6175466; doi:10.1002/path.5130)
Supplement: Supplementary file 1 — Supplementary materials and methods [file PATH-246-180-s001.docx]

**Supplementary materials and methods**

***Cell lines, Chemicals, Pharmacological inhibitors and Transfection***

The NPC cell line C666-1 and HK-1 were maintained in RPMI 1640 supplemented with 10% FBS. The SV40 large T-immortalized nasopharyngeal epithelial cell line NP69 was maintained in keratinocyte-serum free medium supplemented with 0.2 ng/ml EGF and 30 μg/ml bovine pituitary extract (Thermo Fisher Scientific, Waltham, MA USA). NP69-pLNSX, NP69-LMP1, HK1-pLNSX and HK1-LMP1 are stable cell lines generated after retroviral transduction with either pLNSX (Control) or pLNSX-LMP1 (LMP1) and G418 selection. Torin 1, Torin 2, luteolin and fatostatin (Abcam, Cambridge UK) were dissolved in DMSO and diluted in medium immediately prior to use. The plasmids used for transient transfection included pCDNA3 (empty vector) and pCDNA3-LMP1.

***DNA constructs and siRNA***

The scrambled shRNA control and LMP shRNA vectors were generated by inserting a fragment of synthesized oligo with a scrambled sequence or a sequence from LMP1 into the pSUPER.retro.puro vector (oligoengine). The sequence of scrambled shRNA is CGT GAT CTT CAC CGA CAA GAT. The sequence of LMP1 shRNA 1 is GGA ATT TGC ACG GAC AGG C and LMP1 shRNA 2 is GCT CAT CGC TCT CTG GAA T. The pGL2-3xSRE luciferase vector was obtained from ATCC (Manassas, VA, USA). pGL2-3xSRE is a reporter construct containing three tandem copies of an SRE/SP1 element (Cell 1996 87:415-426). pGL3-FASN (a luciferase reporter containing promoter sequence of FASN) was kindly provided by Dr. Qiang Liu, University of Saskatchewan, Canada [15]. siRNAs targeting LMP1, control siRNA, and SMARTpool: ON-TARGETplus siRNAs targeting mTOR, Raptor, Rictor were purchased from Dharmaon Inc., Lafayette, CO, USA. The target sequences of LMP1 siRNA (AAC UGG UGG ACU CUA UUG G & GGA AUU UGC ACG GAC AGG C) were obtained from Marquitz et al (PNAS 2012 109: 9593-9598). Transient transfection of siRNA (100 pmol) or DNA was performed using Lipofectamine 2000 (Thermo Fisher Scientific) or Fugene HD (Promega, Madison, WI, USA) respectively, according to manufacturer’s instructions.

***Western Blotting Analysis***

To avoid the effects of serum on SREPB1 activation and SREBP1-mediated lipogenesis, culture cells were maintained in serum-free medium for 16 h prior to protein extraction with RIPA buffer. Total cell lysates (5–50 μg of protein) were separated by SDS-PAGE (10% or 4–12% gels) and transferred to a PVDF membrane prior to immunoblotting. Mouse monoclonal antibodies to LMP1 (clones CS1-4; Cat. No. M0897) were purchased from Dako (Glostrup, Denmark), and α-tubulin (Cat. No. sc-8035) from Santa Cruz Biotechnology (Dallas, TX, USA). The rabbit polyclonal antibody to phospho-SREBP1 (S439) was obtained from Abcam (Cat. No. ab138663) Cambridge, UK). The mouse monoclonal [2A4] antibody to SREBP1 was purchased from Abcam (Cat. No. ab3259) and Santa Cruz Biotechnology (Cat. No. sc-13551). The rabbit monoclonal antibody to FASN (Cat. No.: 3180), mTOR (Cat. No. 2983), Raptor (Cat. No. 2280), Rictor (Cat. No. 2114), phospho-ACLY (Ser455) (Cat. No. 4331), phospho-p70S6K (Ser371) (Cat.No. 9208) and phospho-4EBP1 (Thr37/46) (Cat.No. 2855) were purchased from Cell Signaling Technology (Beverley, MA, USA).

***Quantitative -PCR***

All qPCR products were obtained using Power SYBR green PCR Master Mix Kit (Thermo Fisher Scientific) and the following primers: *LMP1* Forward ATCTCCTTTGGCTCCTCCTGTT, Reverse CTGCCCTCGTTGGAGTTAGAGT. *SREBF1a* Forward CGGCTGCATTGAGAGTGAA, Reverse TCCCCATCCACGAAGAAAC. *SREBF1c* Forward AAACTGCGCCAGGAGAACA, Reverse GGGGGACATCAGAAGGACA. *FASN* Forward AGGTGTCCACCAGCAACATC, Reverse ACTCTGGGGTCTGGTTCTCC. *TBP* Forward GCACAGGAGCCAAGAGTGAA, Reverse CATCACAGCTCCCCACCA

The values of the relative quantification were calculated by the CT method. Data are shown with the value of the reference sample set at 1.

***Immunofluorescence staining***

Cells seeded on culture slides (Falcon, BD Biosciences) were incubated in serum-free medium for 12–16 h. After fixation in 4% paraformaldehyde and permeabilization with 0.2% Tween 20, cells were blocked with 1% BSA and 10% heat-inactivated normal goat serum. After blocking, cells were incubated with a 1:200 dilution of the FASN antibody (Cat No.: 610962, BD BIosciences, Cambridge, UK) at room temperature for 2 h and then incubated with Alexa Fluor 488-conjugted goat anti-mouse antibody (Cat No. A11001, Molecular Probes, Life Technologies) for 1 h. After washing with PBS, slides were mounted with Prolong Antifade solution with DAPI (Thermo Fisher Scientific). Images were captured with a Zeiss inverted fluorescence microscope.

***Lipid Droplets fluorescence staining***

Nile Red fluorescence staining was performed with Lipid Droplets Fluorescence Assay Kit according to the manufacture protocol (Cayman Chemical, Ann Arbor, MI, USA). In brief, 4–10x10^3^ cells were plated into black sided clear bottom 96-well-microplates. One day before staining assay, cells were incubated in serum free medium. As a positive control, cells in completed medium were treated overnight with Oleic Acid provided from assay kit at 1:2000 dilution. For lipid droplets staining, cells were fixed with 1X assay fixative, washed with PBS and then stained with working solution of Hoechst 33342 (1 μg/ml)) and Nile Red (1:1000). The fluorescence of cells were determined using a VICTOR X3 multilabel plate reader (PerkinElmer, Waltham, MA). Hoechst 33342 fluorescence was measured with an excitation of 355 nm and an emission of 460 nm, while Nile Red fluorescence was determined using a 485 nm excitation and 535 nm emission. Differences in cell number were corrected by using Hoechst 33342 fluorescence signal to normalize the Nile Red signal in each well. Data represents the mean of 8 determinations. Cell images were taken by automated Zeiss^TM^ Axiovert Z1 microscope (Zeiss, Jena, Germany).

***Immunohistochemical staining***

# For the immunohistochemical staining (IHC) study, 38 archival formalin-fixed paraffin-embedded NPC primary tumors were recruited from the Department of Anatomical and Cellular Pathology at Prince of Wales Hospital, Hong Kong. The study protocol was approved by the Joint CUHK–NTE Clinical Research Ethics Committee, Hong Kong. All specimens were taken before treatment and were histologically evaluated to be EBV-positive non-keratinizing nasopharyngeal carcinomas, as demonstrated by EBER *in situ* hybridization. The primary antibodies used in this study were the anti-LMP1 monoclonal antibody (CS1-4) (1:100 dilution; Cat. No. M0897, DAKO) and anti-FASN antibody (1:200 dilution; Cat. No. 3189, Cell Signaling). The expression of FASN was scored based on staining intensity and proportion. The intensity score was determined according to the average staining intensity of tumor cells (0, none; 1, weak; 2, intermediate; 3, strong). The proportion score was based on the proportion of tumor cells with positive staining (0, none; 1, <=10%; 2, 10-25%; 3, >25-50%; 4, >50%). For each specimen, at least 200 tumor cells were counted. The immunoactivity score is the product of intensity score and proportion score, ranging from 0 to 12. The protein expression was categorized into absence/low (score 0-2) intermediate (score 3-5), and high (score 6-9). For IHC study in xenografts, cancer tissues were de-paraffinized, rehydrated, and subjected to pressure cooking for antigen retrieval, and then blocking in 1% BSA prior to antibody staining. The rabbit monoclonal antibody to FASN (1:400 dilution; Cat. No. 3189) and cleaved capase-3 (1:200 dilution, Cat. No. 9561) were purchased from Cell Signaling. The rabbit monoclonal antibody to Ki67 (1:400 dilution; MA5-14520) were from Thermo Fisher Scientific.

***Cell Proliferation Assay***

The cell proliferation reagent CCK-8 was purchased from Dojindo Molecular Technologies (Rockville, MD, USA). In routine assays, 5x10^3^ cells in 100 μl of medium were seeded into 96-well plates in triplicate. The CCK-8 assay was performed as follows. Briefly, 10 μl of CCK-8 reagent to each well and cells incubated at 37 °C for 4 h. The absorbance was then measured at 450 nm. Each sample was performed in triplicate. The results are presented as relative growth rate by dividing the absorbance value of the cells at indicated time points by the absorbance value of the cells 1 day after cell plating. Each data point is represented by the mean and SD.

**De novo *Lipogenesis assay***

Cells grown in 6-well plates, were incubated in serum-free medium containing 2.5 μCi/ml [1-^14^C] acetate (42 μM final concentration, Perkin Elmer) for 8–10 h. After washing twice with PBS, cells were lysed in 200 μl 0.5% Triton X-100/PBS. Lipids were extracted by successive addition of 200μl methanol and 400 μl chloroform, following by centrifugation. The organic phase was then transferred to a new tube for air drying. The dried lipid was re-suspended in 50 μl chloroform for scintillation counting. Results were normalized to total protein content. The results are presented as relative lipogenesis and data are shown with the value of the reference sample set at 1.

**In vivo *Tumorigenicity Experiments***

To examine the anti-tumor effects of lipogenesis inhibitors *in vivo*, C666-1 cells (1×10^7^) were subcutaneously inoculated into the flanks of female BALB/c nude mice (nu/nu) (3-6 weeks old). All mice were anesthetized with 2,2,2-tribromoethanol (Avertin) prior to tumor cell inoculation. When tumor size reached an average size of approximately 50 mm^3^, animals were randomly assigned to different experimental groups and intravenously injected with either PBS (vehicle), luteolin (20 mg/kg) or fatostatin (15 mg/kg) every 2-3 days for 3 weeks. Body weight and tumor size was measured every 2-3 days. At the end point, animals were killed by cervical dislocation and tumors were harvested for weight measurement and for further analysis. The study protocol was approved by the University Animal Experimentation Ethics Committee (AEEC), CUHK, and the animal license was obtained from the Hong Kong Government, Department of Health.
