# Supplementary material for: Activation of sterol regulatory element‐binding protein 1 (SREBP1)‐mediated lipogenesis by the Epstein–Barr virus‐encoded latent membrane protein 1 (LMP1) promotes cell proliferation and progression of nasopharyngeal carcinoma
Source: J Pathol. 2018 Aug 22;246(2):180–90. doi: 10.1002/path.5130 (PMC6175466; doi:10.1002/path.5130)
Supplement: Supplementary file 2 — Figure S1. Silencing of LMP1 leads to a reduction of FASN promoter activity. C666‐1 were transfected with either a negative control siRNAs (SiCtl) or LMP1 siRNA (siLMP1) together with the pGL3‐FASN luciferase promoter vector. After incubation in serum‐free medium for 12 h, cells were harvested for luciferase analysis. Luciferase activity was normalized to Renilla activity and was plotted relative to the siRNA control (SiCtl) (set at 1). Figure S2. The inhibitory effects of Luteolin and Fatostatin on SREBP1 maturation and FASN expression. Western blotting analysis of SREBP1 and FASN in LMP1 expressing NP69 and HK‐1 nasopharyngeal epithelial cells treated with luteolin or fatostatin. Figure S3. Body weight of mice bearing C666‐1 NPC xenografts during the course (19 days) of treatment with PBS (control), luteolin (20 mg/kg), fatostatin (20 mg/kg). Figure S4. The inhibitory effects of Luteolin and Fatostatin on FASN expression, cell proliferation and apoptosis in NPC xenografts. Western blotting analysis of FASN, cleaved PARP and Cleaved Caspase 3 proteins in NPC tumours harvested from animals treated with PBS, (A) luteolin or (B) fatostatin. Figure S5. The mechanism of LMP1 in upregulation of lipogenesis. Induction of mTOR by LMP 1 increases/activates SREBP1‐meditated lipogenesis, facilitating cell proliferation and NPC tumour growth. [file PATH-246-180-s003.pdf]

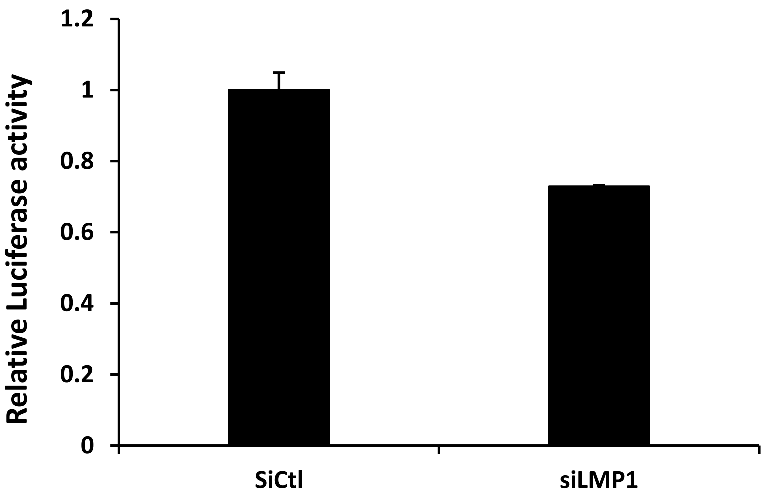


**Supplementary Figure S1. Silencing of LMP1 leads to a reduction of *FASN* promoter activity.** C666-1 were transfected with either a negative control siRNAs (SiCtl) or LMP1 siRNA (siLMP1) together with the pGL3-FASN luciferase promoter vector. After incubation in serum-free medium for 12 h, cells were harvested for luciferase analysis. Luciferase activity was normalized to Renilla activity and was plotted relative to the siRNA control (SiCtl) (set at 1).

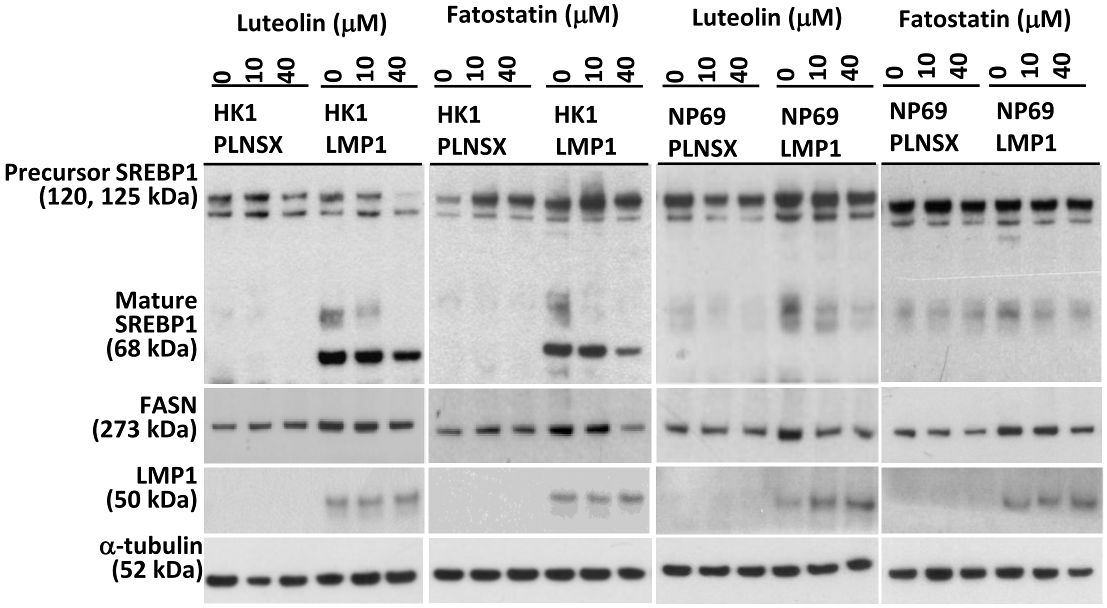


**Supplementary Figure S2. The inhibitory effects of Luteolin and Fatostatin on SREBP1 maturation and FASN expression.** Western blotting analysis of SREBP1 and FASN in LMP1 expressing NP69 and HK-1 nasopharyngeal epithelial cells treated with luteolin or fatostatin.

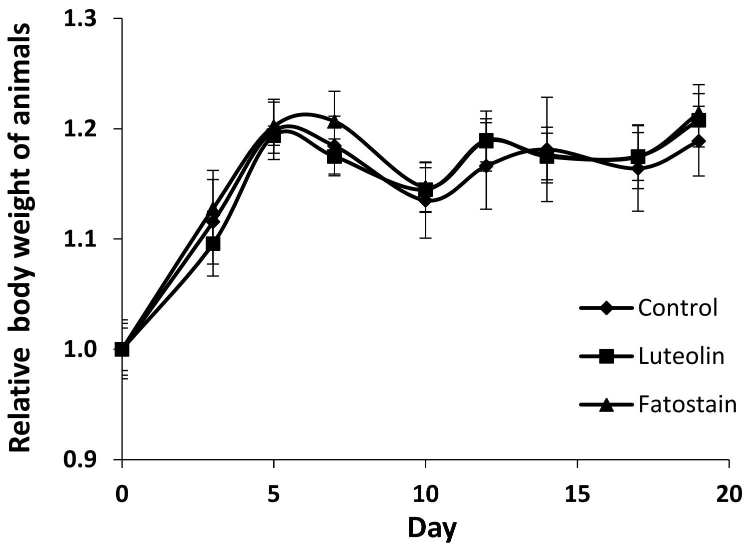


**Supplementary Figure S3**. Body weight of mice bearing C666-1 NPC xenografts during the course (19 days) of treatment with PBS (control), luteolin (20 mg/kg), fatostatin (20 mg/kg).

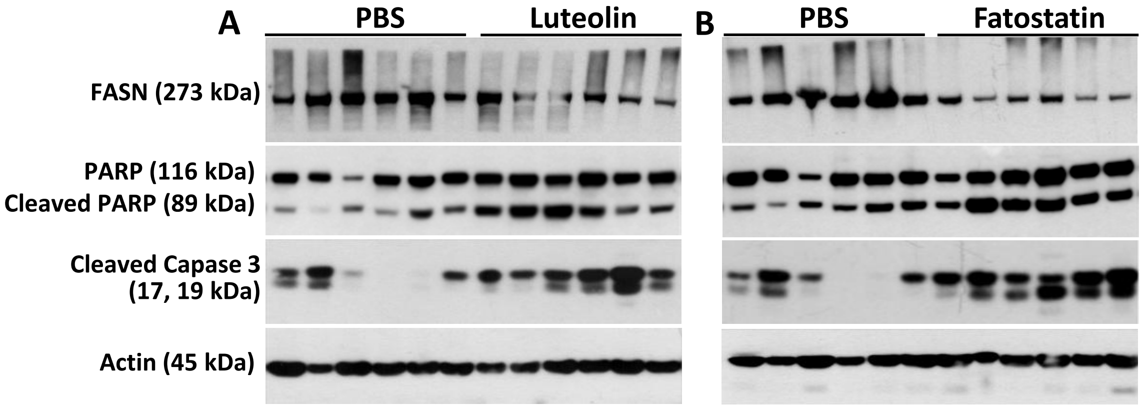


**Supplementary Figure S4. The inhibitory effects of Luteolin and Fatostatin on FASN expression, cell proliferation and apoptosis in NPC xenografts.** Western blotting analysis of FASN, cleaved PARP and Cleaved Caspase 3 proteins in NPC tumours harvested from animals treated with PBS, (A) luteolin or (B) fatostatin.

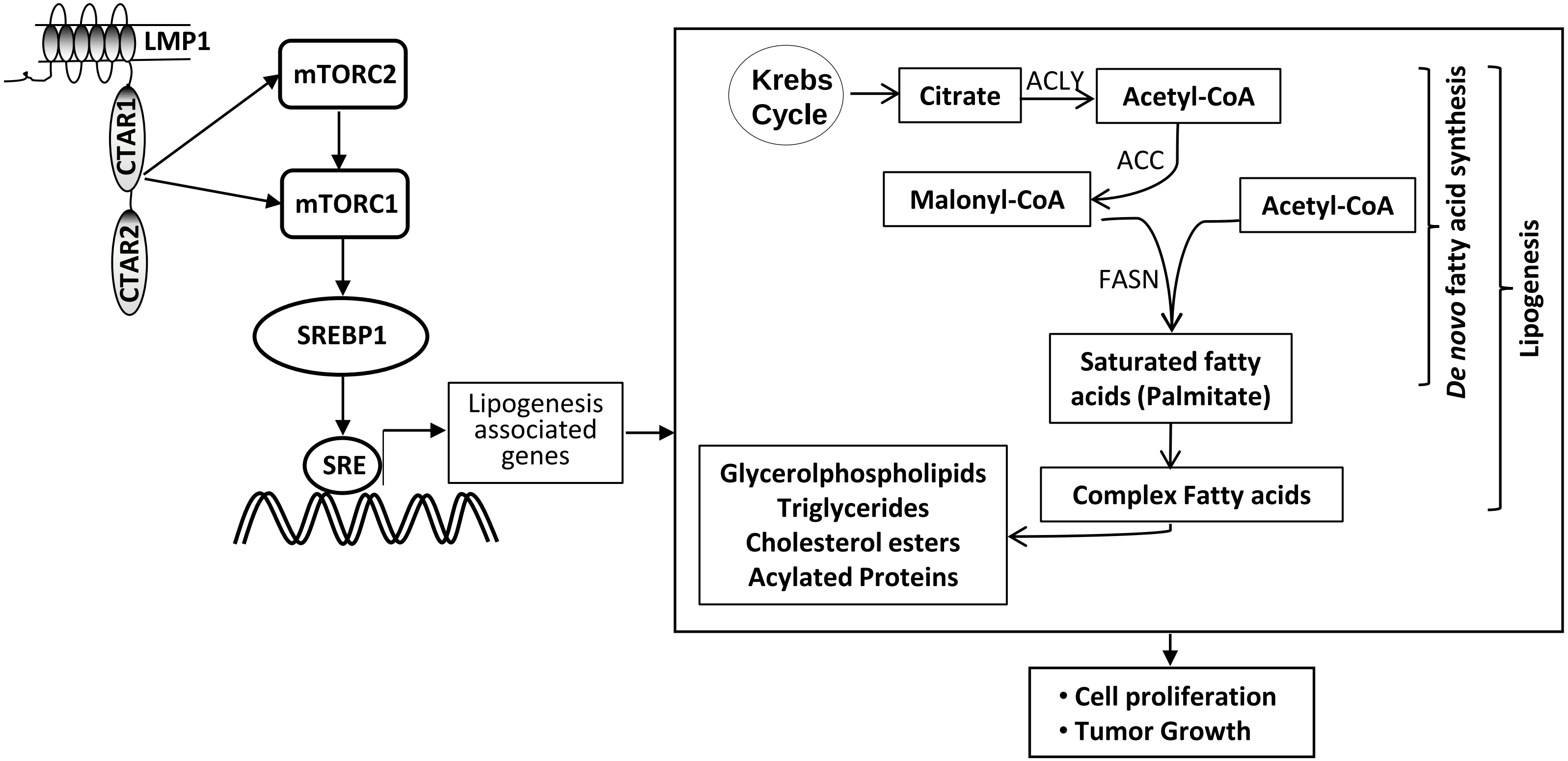


**Supplementary Figure S5. The mechanism of LMP1 in upregulation of lipogenesis.** Induction of mTOR by LMP1 increases/activates SREBP1-meditated lipogenesis, facilitating cell proliferation and NPC tumour growth.
