# Supplementary material for: Activation of sterol regulatory element‐binding protein 1 (SREBP1)‐mediated lipogenesis by the Epstein–Barr virus‐encoded latent membrane protein 1 (LMP1) promotes cell proliferation and progression of nasopharyngeal carcinoma
Source: J Pathol. 2018 Aug 22;246(2):180–90. doi: 10.1002/path.5130 (PMC6175466; doi:10.1002/path.5130)
Supplement: Supplementary file 3 — Table S1. Clinicopathologic characteristics of patients with NPC [file PATH-246-180-s002.docx]

**Supplementary Table S1. Clinicopathologic characteristics of patients with NPC**

| **Parameters** | **Category** | **FASN Expression** | | ***p* value** |
| --- | --- | --- | --- | --- |
|  |  | **Low (<3)** | **High (≥3)** |  |
| **Gender** | Male | 13 | 18 | 1 |
|  | Female | 3 | 4 |  |
| **Age (years)** | >53** | 11 | 11 | 0.336 |
|  | ≤53 | 5 | 11 |  |
| **Primary Tumor (T)** | T1-T2 | 11 | 4 | 0.026* |
|  | T3-T4 | 5 | 18 |  |
| **Nodal Status (N)** | N0 | 6 | 0 | 0.005* |
|  | N1-N3 | 9 | 22 |  |
| **Stage** | I-II | 5 | 3 | 0.243 |
|  | III-IV | 11 | 19 |  |

**Mean age

*Statistically significant
